# Clinical Benefits of new Systemic Therapy for Small‐Cell Lung Cancer Over Two Decades: A Cross‐Sectional Study

**DOI:** 10.1111/crj.70032

**Published:** 2024-10-30

**Authors:** Yuejing Chen, Honghong Liu, Shaohua Bai, Xuejiao Han, Fei Jin, Bo Cui

**Affiliations:** ^1^ Department of Pulmonary and Critical Care Medicine Xingtai Third Hospital Xingtai Hebei China

**Keywords:** clinical benefit, ESMO‐MCBS, lung cancer, randomized controlled trials

## Abstract

**Introduction:**

Small cell lung cancer (SCLC) is one of the most lethal malignancies worldwide. This study aimed to examine the clinical benefits of new systemic therapies derived from randomized controlled trials (RCTs) published from 2002 to 2023 based on the magnitude of clinical benefit scale developed by the European Society for Medical Oncology (ESMO‐MCBS).

**Methods:**

We searched PubMed for Phase 3 RCTs on systemic therapy for SCLC published between January 2002 and December 2023. Therapeutic benefit was graded from 5 to 1 according to the ESMO‐MCBS framework, with a score of 4 or 5 representing a meaningful clinical benefit. The statistical power of the trial design was also assessed using ESMO‐MCBS.

**Results:**

Sixty‐four RCTs with 23 683 participants were eligible for inclusion. The number of RCTs related to molecular targeted therapy or immunotherapy has increased over the years. Among the 62 RCTs for which statistical power could be evaluated, 38 (61.3%) were designed to identify an effect size that would meet the ESMO‐MCBS benefit threshold and were less likely to investigate second‐ or subsequent‐line treatment (15.8% vs. 50.0%, *p* = 0.004), have noninferiority design (0% vs. 25.0%, *p* = 0.002) and set PFS (0% vs. 16.7%) or response rate (0% vs. 16.7%) as the only primary endpoint (*p* = 0.002). The ESMO‐MCBS framework was applied in 29 RCTs reporting positive results, and only 8 (27.6%) met the threshold for a clinical benefit. The RCTs designed to detect differences that would meet the thresholds were more likely to demonstrate meaningful clinical benefit (87.5% vs. 50.0%, *p* = 0.099).

**Conclusion:**

Most positive SCLC‐RCTs did not meet the ESMO‐MCBS threshold for meaningful clinical benefits. Strict power calculations should be adopted in the design of future RCTs.

## Graphical abstract

Over the past 20 years, most of the new systemic therapies for **s**mall‐cell lung cancer did not have a meaningful clinical benefit.

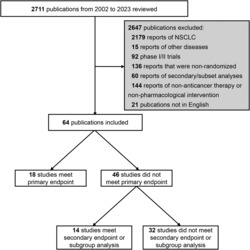

## 1 Introduction

Globally, lung cancer remains the leading cause of cancer‐related deaths [1]. Small‐cell lung cancer (SCLC) is the most lethal subtype of lung cancer, accounting for 13%–15% of all cases, and is characterized by rapid proliferation and a high metastatic potential [2]. Even with active treatment, the median overall survival (OS) is usually less than 2 years for limited‐stage SCLC and less than 1 year for extensive‐stage SCLC [3]. Platinum‐based chemotherapy is the standard first‐line systemic treatment of SCLC [4]. In contrast to nonsmall cell lung cancer (NSCLC), limited progress has been made in SCLC treatment over the past decades. There is a substantial demand for effective treatment options to prolong the survival of patients with SCLC.

Randomized controlled trials (RCTs), representing the highest level of evidence‐based medicine, have led to significant therapeutic advancements in oncology. With the rapid growth in the number of RCTs, the assessment of the clinical benefits of new therapies has become a great concern. Patients and some care providers may only be interested in whether an RCT has achieved statistical significance, while having a limited understanding of the extent of the benefits.

In fact, “meaningful clinical benefit” can be summarized as living longer and/or living better, that is, improved OS and/or quality of life (QoL). Minor improvements in therapeutic endpoints may offer minimal benefits, particularly for surrogate endpoints, such as progression‐free survival (PFS) [5, 6]. To quantify the clinical benefits of anticancer therapies, the European Society for Medical Oncology (ESMO) published the magnitude of the clinical benefit scale (ESMO‐MCBS) in 2015 [7] and revised it in 2017 [8]. Previous studies have adopted this tool and have shown that a significant proportion of new therapies have failed to provide meaningful benefits for patients with NSCLC [9, 10, 11]. In the current study, we aimed to examine the clinical outcomes obtained from RCTs on systemic therapies for SCLC published over the past 20 years using the ESMO‐MCBS framework.

## 2 Materials and Methods

### 2.1 Search Strategy and Selection Criteria

A systematic search of Phase 3 RCTs on systemic therapy for SCLC was conducted using the PubMed database on April 16, 2024. The following terms were used in the search: Small‐cell Lung [Title/Abstract] AND (Cancer OR Carcinoma) AND Randomized Controlled Trial [Publication Type]. The “Results by Year” filter was used to select publications between 2002 and 2023. Eligible trials were Phase 3 RCTs, which compared at least two arms of drug therapies in patients with SCLC. RCTs that compared different administration routes, doses, or timings of the same regimen were eligible for inclusion. Phases 1 and 2 trials were excluded because they were typically preliminary assessments of safety and efficacy prior to Phase 3 trials and were seldom designed to document a meaningful clinical benefit. Other exclusion criteria were as follows: reports on NSCLC or other diseases, nonrandomized trials, secondary or subset analyses from RCTs, nonanticancer therapy or nonpharmacological intervention, and non‐English reports.

### 2.2 Data Extraction and Management

Two authors reviewed the search results and selected the eligible studies using a two‐step approach. First, the title and abstract of each article were reviewed to exclude trials that were clearly unsuitable based on predetermined eligibility criteria. Second, the main texts of the remaining articles were reviewed to eliminate trials that met the exclusion criteria. Subsequently, a data abstraction form was created to document the trial design, end points, conclusions, and corresponding supportive information. Notably, for trials with more than one experimental arm, the arm used for assessment achieved the best results for the primary endpoint. When multiple publications of the same RCT were available, the latest report was used for the assessment. We also performed a PubMed search for updated data on survival and QoL as outlined in the original article.

Author conclusions were rated on a Likert scale of 1 to 7, with scores determined after initially reviewing the abstract conclusion: ratings of 1–3 favored the control arm, ratings of 5–7 favored the experimental arm, and ratings of 4 indicated equivalence. Ratings of 6–7 was considered as “strong endorsement” [12]. The 2023 journal impact factors (IFs) were gathered from Journal Citation Reports released by Clarivate Analytics.

### 2.3 ESMO‐MCBS Scoring

Two authors conducted the assessments, and any disagreement was resolved by consensus. ESMO‐MCBS grading was applied to RCTs that demonstrated a statistically significant advantage for the experimental arm, either in the primary endpoint analysis or in the secondary endpoint/pre‐specified subgroup analysis. Noninferiority RCTs were available for assessment if they met the noninferiority threshold. According to the ESMO‐MCBS framework [7, 8], Forms 2a and 2b were assessed by considering the absolute gain in overall survival (OS) and/or progression‐free survival (PFS) and the lower limit of the 95% confidence interval (CI) of the corresponding hazard ratio (HR). Any significant improvement or deterioration in toxicity or QoL could modify the ESMO‐MCBS grade. More details are provided in Table S1. For noninferior RCTs, Form 2c was applied, considering toxicity or QoL data. Each trial was rated from 5 to 1, with scores of 5 or 4 indicating significant clinical benefits. Examples are provided in Table S2.

We also applied the ESMO‐MCBS framework to the estimated effect size (as described in the sample size calculation for each RCT) to detect whether an RCT was designed to detect a difference that would meet the benefit threshold. As the 95% CI of the targeted HR and other relevant information were not reported, only the estimated absolute gain (e.g., OS gain) was considered for scoring without any adjustments. Similarly, a minimal grade of 4 represents RCTs powered to identify an effect size that would meet the thresholds.

### 2.4 Statistical Analysis

The primary outcome was the proportion of RCTs that met the ESMO‐MCBS thresholds for clinically meaningful benefit. Secondary outcomes included (i) temporal trends from 2002 to 2023 and (ii) the proportion of RCTs that were designed to identify an effect size that would meet the threshold for clinical benefit. Trends over time of the number of RCTs were plotted, and by visual inspection of the graphs, we grouped the experimental therapies into an increasing or decreasing trend.

Categorical variables were reported as frequencies (percentages) and compared using the chi‐square test or Fisher's exact test. Continuous variables were reported as medians (Q1–Q3) and compared using the Mann–Whitney *U* test. Statistical significance was defined as a two‐sided *p* value of <0.05. All statistical analyses were performed using SPSS version 22 software (SPSS Inc., Chicago, IL, United States).

## 3 Results

### 3.1 Trial Characteristics

**FIGURE 1:**
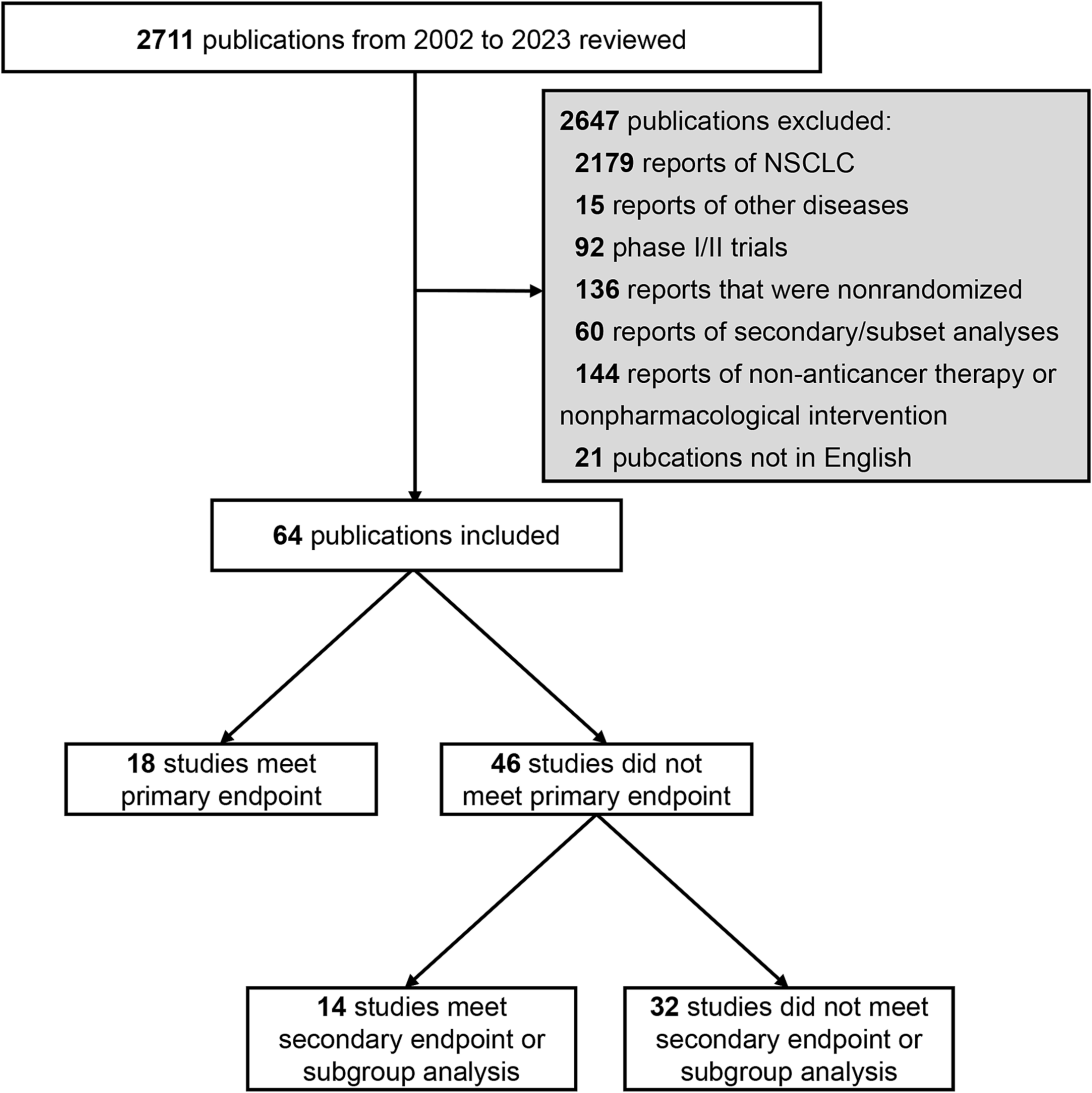
Flow diagram depicting the trial selection process for the review.

Our PubMed search yielded 2711 articles. After excluding 2647 ineligible articles, 64 RCTs with 23 683 (median, 305) participants were included in the analysis (Figure 1). Among these RCTs, 57 (89.1%) enrolled patients with extensive‐stage SCLC and 46 (71.9%) investigated first‐line strategies. Most RCTs had superior designs (90.6%) and set OS as the primary or coprimary endpoint (87.5%); however, only 20 RCTs (31.3%) set QoL as a secondary endpoint. Chemotherapy was the most common experimental treatment, with 43 RCTs (67.2%), of which 34 adopted a platinum‐based regimen (79.1%). Molecular‐targeted therapy (14.1%) and immunotherapy (14.1%) were the second most common experimental arms. Seven of the nine RCTs related to targeted therapy (77.8%) investigated second‐line or maintenance therapies. Eight of the nine RCTs related to immunotherapy (88.9%) targeted programmed cell death 1 (PD‐1) or its ligand (PD‐L1).

### 3.2 Temporal Trends of the RCTs

**FIGURE 2:**
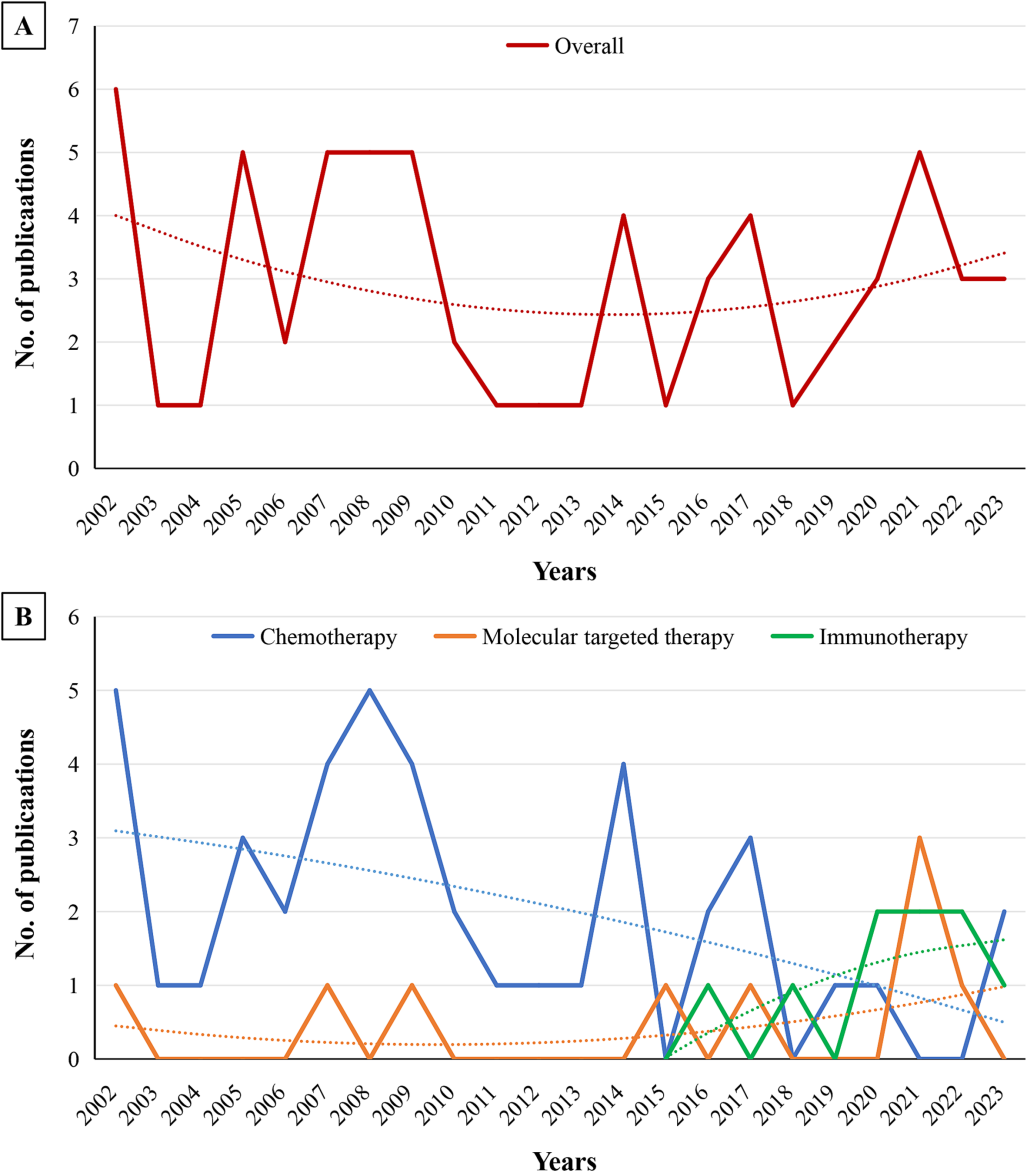
Number of randomized controlled trials of systemic therapy for small cell lung cancer published during 2002–2023.

In general, the number of published RCTs on SCLC decreased annually before 2013 and increased thereafter (Figure 2A). When stratified by therapy, the proportion of chemotherapy related RCTs showed a downward trend (85.3% in 2002–2012 vs. 46.7% in 2013–2023), whereas the proportions of RCTS related to targeted therapy (8.8% in 2002–2012 vs. 20.0% in 2013–2023) and immunotherapy (0% in 2002–2012 vs. 30.0% in 2013–2023) showed an upward trend (Figure 2B and Table 1). Compared to RCTs published before 2013, those published after 2013 were more likely to come from Asian countries (36.7% vs. 8.8%, *p* = 0.014) and receive industry funding (90.0% vs. 64.7%, *p* = 0.020; Table 1).

**TABLE 1:** Characteristics of all randomized clinical trials.

Characteristics, *n* (%)	Total (*n* = 64)	2002–2012 (*n* = 34)	2013–2023 (*n* = 30)	*p*
Stage				0.286
Extensive stage	34 (53.1)	15 (44.1)	19 (63.3)	
Limited stage	7 (10.9)	4 (11.8)	3 (10.0)	
Both types	23 (35.9)	15 (44.1)	8 (26.7)	
Setting				0.153
First line	46 (71.9)	27 (79.4)	19 (63.3)	
Second or subsequent line	18 (28.1)	7 (20.6)	11 (36.7)	
Design				1.000
Superiority	58 (90.6)	31 (91.2)	27 (90.0)	
Noninferiority	6 (9.4)	3 (8.8)	3 (10.0)	
Therapy				0.003
Cytotoxic	43 (67.2)	29 (85.3)	14 (46.7)	
Small molecule inhibitor	9 (14.1)	3 (8.8)	6 (20.0)	
Antibody	1 (1.6)	1 (2.9)	0 (0.0)	
Immunogenic	9 (14.1)	0 (0.0)	9 (30.0)	
Hormonal	2 (3.1)	1 (2.9)	1 (3.3)	
Primary endpoint				0.142
OS + PFS	6 (9.4)	1 (2.9)	5 (16.7)	
OS only	50 (78.1)	30 (88.2)	20 (66.7)	
PFS only	4 (6.3)	2 (5.9)	2 (6.7)	
Other	4 (6.3)	1 (2.9)	3 (10.0)	
Sample size				0.567
Median (Q1–Q3)	305 (203–528)	295 (216–456)	331 (187–573)	
Study origin				0.014
Non‐Asian	50 (78.1)	31 (91.2)	19 (63.3)	
Asian	14 (21.9)	3 (8.8)	11 (36.7)	
Multinational study				0.344
Yes	28 (43.8)	13 (38.2)	15 (50.0)	
Industry sponsorship				0.020
Present	49 (76.6)	22 (64.7)	27 (90.0)	

The median OS of the experimental arm was reported in all RCTs (Figure S1). When stratified by therapy, the global median OS was significantly shorter for extensive disease versus limited disease (10.2 [8.5–11.9] vs. 25.0 [17.0–27.2] months). For limited disease, no significant breakthroughs occurred after the establishment of concurrent chemotherapy with etoposide‐platinum and thoracic radiotherapy [13]. In patients with extensive disease, median OS was significantly prolonged after the introduction of PD‐1/PD‐L1 inhibitors [14, 15].

### 3.3 Clinical Benefit Analyses

Among the 62 RCTs available on the ESMO‐MCBS score, 38 (61.3%) were designed to identify an effect size that would meet the threshold for clinical benefit. RCTs designed to meet this threshold were less likely to investigate second‐ or subsequent‐line treatment (15.8% vs. 50.0%, *p* = 0.004), have noninferiority design (0% vs. 25.0%, *p* = 0.002), and set PFS (0% vs. 16.7%) or response rate (0% vs. 16.7%) as the only primary endpoint (*p* = 0.002; Table 3).

We then applied the ESMO‐MCBS framework to the results of the 29 RCTs that showed a statistically significant advantage in the experimental group. Among these, 18 RCTs were scored based on OS data (Form 2a), 7 based on PFS data (Form 2b), and 4 based on toxicity/QoL and response data (Form 2c). Overall, 8 RCTs (27.6%) scored 4 and met the ESMO‐MCBS benefit threshold: 6 (20.7%) scored 3; 3 (10.3%) scored 2; and 12 (41.4%) scored 1 (Table 2). The RCTs designed to detect differences that would meet the benefit thresholds were more likely to demonstrate meaningful clinical benefit (87.5% vs. 50.0%, *p* = 0.099; Table 3).

**TABLE 2:** Characteristics of the positive trials and their corresponding final ESMO‐MCBS scores.

Medication	Setting	Positive endpoint	Endpoint assessment	Adjustment	ESMO‐MCBS
Irinotecan plus cisplatin versus etoposide plus cisplatin [16]	First line	OS	OS_gain_ = 3.4 m (OS_control_ ≤ 12 m) HR_lower limit of 95% CI_ < 0.65	None	4 (Form 2a)
Etoposide versus observation [17]	Maintenance	PFS (secondary)	PFS_gain_ = 1.73 m (PFS_control_ > 6 m) HR_lower limit of 95% CI_ < 0.65	Fail to improve OS (down 1)	2 (Form 2b)
Concurrent versus sequential chemoradiotherapy [13]	First line	OS	OS_gain_ = 7.5 m (OS_control_ 12–24 m) HR_lower limit of 95% CI_ < 0.70	None	4 (Form 2a)
Etoposide and cisplatin versus cyclophosphamide, epirubicin, and vincristine [18]	First line	OS	OS_gain_ = 2.4 m (OS_control_ ≤ 12 m) HR_lower limit of 95% CI_ < 0.65	None	3 (Form 2a)
Paclitaxel, etoposide, and carboplatin versus carboplatin, etoposide, and vincristine [19]	First line	OS	OS_gain_ = 0.8 m (OS_control_ ≤ 12 m)	Less SAE (up 1)	2 (Form 2a)
Ifosfamide, carboplatin, etoposide, and vincristine versus cisplatin plus etoposide [20]	First line	OS	OS_gain_ = 4.0 m (OS_control_ ≤ 12 m) HR_lower limit of 95% CI_ < 0.65	None	4 (Form 2a)
Topotecan versus observation [21]	Maintenance	OS	OS_gain_ = 2.8 m (OS_control_ ≤ 12 m) HR_lower limit of 95% CI_ < 0.65	Improved QoL (up 1)	4 (Form 2a)
Standard versus dose‐intensified chemotherapy [22]	First line	OS	OS_gain_ = 11.8 m (OS_control_ 12–24 m) HR_lower limit of 95% CI_ < 0.70	None	4 (Form 2a)
Oral versus intravenous topotecan [23]	Second line	ORR	ORR_gain_ = −3.6% (noninferiority)	None	1 (Form 2c)
Thalidomide versus placebo [24]	Second line	OS (Subgroup)	2‐y OS_gain_ ≥ 10%	More SAE (down 1)	3 (Form 2a)
Irinotecan plus carboplatin versus etoposide plus carboplatin [25]	First line	OS	OS_gain_ = 1.4 m (OS_control_ ≤ 12 m)	None	1 (Form 2a)
Gemcitabine plus carboplatin versus cisplatin and etoposide [26]	First line	QoL (Secondary)	Less symptoms	None	3 (Form 2c)
Topotecan plus cisplatin versus etoposide plus cisplatin [27]	First line	ORR	ORR_gain_ = 10.0% (noninferiority)	None	1 (Form 2c)
Radiotherapy with first‐ versus third‐cycle chemotherapy [28]	First line	ORR	ORR_gain_ = 2.0% (noninferiority)	None	1 (Form 2c)
Amrubicin versus topotecan [29]	Second line	OS (Subgroup)	OS_gain_ = 0.5 m (OS_control_ ≤ 12 m)	None	1 (Form 2a)
Cisplatin, etoposide, and irinotecan versus topotecan [30]	First line	OS	OS_gain_ = 5.7 m (OS_control_ 12–24 m) HR_lower limit of 95% CI_ < 0.70	None	4 (Form 2a)
Ipilimumab plus etoposide and platinum versus placebo plus etoposide and platinum [31]	First line	PFS (Secondary)	HR_lower limit of 95% CI_ > 0.65	Fail to improve OS (down 1)	1 (Form 2b)
Cisplatin plus etoposide with versus without bevacizumab [32]	First line	PFS (Secondary)	PFS_gain_ = 1.0 m (PFS_control_ ≤ 6 m) HR_lower limit of 95% CI_ < 0.65	Fail to improve OS (down 1)	1 (Form 2b)
Irinotecan plus cisplatin versus etoposide plus cisplatin [33]	First line	OS (Subgroup)	OS_gain_ = 1.5 m (OS_control_ ≤ 12 m)	More SAE (down 1)	1 (Form 2a)
Atezolizumab plus chemotherapy versus placebo plus chemotherapy [34]	First line	OS	OSgain = 1.4 m (OScontrol ≤ 12 m)	None	3 (Form 2a)
Pembrolizumab versus placebo plus etoposide and platinum [35]	First line	OS	OS_gain_ = 1.1 m (OS_control_ ≤ 12 m)	None	1 (Form 2a)
Carboplatin plus etoposide versus topotecan [36]	Second line	PFS	PFS_gain_ = 2.0 m (PFS_control_ ≤ 6 m) HR_lower limit of 95% CI_ < 0.65	Fail to improve OS (down 1)	2 (Form 2b)
Durvalumab plus platinum‐etoposide versus platinum‐etoposide [37]	First line	OS	OS_gain_ = 2.4 m (OS_control_ ≤ 12 m) HR_lower limit of 95% CI_ < 0.65	None	3 (Form 2a)
Nivolumab versus chemotherapy [38]	Second line	OS (Subgroup)	PFS_gain_ = 1.9 m (PFS_control_ ≤ 6 m)	Less SAE (up 1)	3 (Form 2a)
Nivolumab and ipilimumab versus placebo [39]	Maintenance	PFS(Secondary)	PFS_gain_ = 0.5 m (PFS_control_ ≤ 6 m) HR_lower limit of 95% CI_ < 0.65	Fail to improve OS (down 1)	1 (Form 2b)
Rovalpituzumab tesirine versus placebo [40]	Maintenance	PFS	PFS_gain_ = 2.6 m (PFS_control_ ≤ 6 m) HR_lower limit of 95% CI_ < 0.65	More SAE (down 1) Fail to improve OS (down 1)	1 (Form 2b)
Niraparib versus placebo [41]	Maintenance	PFS	PFS_gain_ = 0.1 m (PFS_control_ ≤ 6 m) HR_lower limit of 95% CI_ < 0.65	Fail to improve OS (down 1)	1 (Form 2b)
Adebrelimab versus placebo plus carboplatin and etoposide [42]	First line	OS	2‐y OS_gain_ ≥ 10%	None	4 (Form 2a)
Serplulimab versus placebo plus chemotherapy [43]	First line	OS	OS_gain_ = 4.5 m (OS_control_ ≤ 12 m) HR_lower limit of 95% CI_ < 0.65	None	4 (Form 2a)

Abbreviations: CI, confidence interval; HR, hazard ratio; ORR, objective response rate; OS, overall survival; PFS, progression‐free survival; QoL, quality of life; SAE, serious adverse events.

**TABLE 3:** Trial characteristics stratified by whether the trial design or results met the ESMO‐MCBS threshold for clinical benefit.

Characteristics, *n* (%)	Design met the ESMO‐MCBS benefit threshold		*p*	Results met the ESMO‐MCBS benefit threshold		*p*
	Absent (*n* = 24)	Present (*n* = 38)		Absent (*n* = 21)	Present (*n* = 8)	
Year of publication			0.213			0.406
2002–2012	10 (41.7)	22 (57.9)		8 (38.1)	5 (62.5)	
2013–2023	14 (58.3)	16 (42.1)		13 (61.9)	3 (37.5)	
Stage			0.291			0.460
Extensive stage	13 (54.2)	21 (55.3)		13 (61.9)	3 (37.5)	
Limited stage	1 (4.2)	6 (15.8)		1 (4.8)	1 (12.5)	
Both types	10 (41.7)	11 (28.9)		7 (33.3)	4 (50.0)	
Setting			0.004			0.671
First line	12 (50.0)	32 (84.2)		12 (57.1)	6 (75.0)	
Second or subsequent line	12 (50.0)	6 (15.8)		9 (42.9)	2 (25.0)	
Comparison of different dosage			0.136			0.276
Yes	1 (4.2)	7 (18.4)		0 (0.0)	1 (12.5)	
Design			0.002			0.540
Superiority	18 (75.0)	38 (100.0)		18 (85.7)	8 (100.0)	
Noninferiority	6 (25.0)	0 (0.0)		3 (14.3)	0 (0.0)	
Therapy			0.159			0.358
Cytotoxic	16 (66.7)	25 (65.8)		11 (52.4)	6 (75.0)	
Small molecule inhibitor	6 (25.0)	3 (7.9)		4 (19.0)	0 (0.0)	
Antibody	0 (0.0)	1 (2.6)		—	—	
Immunogenic	1 (4.2)	8 (21.1)		6 (28.6)	2 (25.0)	
Hormonal	1 (4.2)	1 (2.6)		—	—	
Primary endpoint			0.002			0.319
OS + PFS	1 (4.2)	4 (10.5)		4 (19.0)	0 (0.0)	
OS only	15 (62.5)	34 (89.5)		14 (66.7)	8 (100.0)	
PFS only	4 (16.7)	0 (0.0)		1 (4.8)	0 (0.0)	
Response rate	4 (16.7)	0 (0.0)		2 (9.5)	0 (0.0)	
Sample size			0.497			0.157
Median (Q1–Q3)	263 (162–607)	319 (220–519)		362 (212–693)	231 (151–447)	
Study origin			0.794			0.164
Non‐Asian	19 (79.2)	29 (76.3)		17 (81.0)	4 (50.0)	
Asian	5 (20.8)	9 (23.7)		4 (19.0)	4 (50.0)	
Multinational study			0.335			0.682
Yes	9 (37.5)	19 (50.0)		11 (52.4)	3 (37.5)	
Industry sponsorship			0.052			0.357
Present	22 (91.7)	27 (71.1)		17 (81.0)	5 (62.5)	
Design to meet the ESMO‐MCBS threshold						0.099^a^
Absent	—	—		10 (50.0)	1 (12.5)	
Present	—	—		10 (50.0)	7 (87.5)	
Author conclusions						0.001
Median (Q1–Q3)	—	—		5 (4.5–6)	7 (6–7)	
Journal impact factor						0.121
Median (Q1–Q3)	—	—		45.3 (20.4–50.5)	48.2 (45.3–103.3)	

^a^ Excluded one unevaluable trial.

### 3.4 Author Conclusions and Journal IFs

Among the 29 RCTs assessed with the ESMO‐MCBS framework, those meeting the benefit threshold were more likely to receive strong endorsements from the authors (100.0% [8/8] vs. 38.1% [8/21], *p* < 0.001; Likert score: 7 [6, 7] vs. 5 [4.5–6], *p* = 0.001). No significant association was found between the journal IF and meeting the threshold for benefits (*p* = 0.121; Table 2).

## 4 Discussion

To the best of our knowledge, this is the first study to examine the true clinical benefits of new anticancer therapies in patients with SCLC based on RCTs published during the past two decades. The key findings include the following: (i) Targeted therapy‐ and immunotherapy‐related trials are increasing, whereas chemotherapy‐related trials are decreasing; however, chemotherapy remains the most frequently investigated therapy; (ii) only 8 of the 64 trials (12.5%) met the ESMO‐MCBS threshold for clinical benefit; and (iii) 38 of the 62 trials (61.3%) were designed to identify an effect size that could meet the criteria for benefit (Table 3).

Systemic chemotherapy serves as the cornerstone treatment for all stages of SCLC. The standard chemotherapeutic regimen for SCLC involves a combination of platinum‐based DNA crosslinking agents (e.g., carboplatin and cisplatin) and topoisomerase inhibitors (e.g., etoposide and irinotecan) [44, 45]. Although the efficacy and safety of this regimen have been extensively demonstrated, most patients experience relapse in the short term after treatment [4]. Therefore, recent trials have focused on combining chemotherapy with other agents or modalities. However, only immunotherapy‐related RCTs have reported positive results for first‐line treatment since 2018 (IMpower133 trial [34]; Figure S1). Due to the substantial heterogeneity and complexity of SCLC, the application of targeted therapies is limited and warrants further exploration [46].

ESMO‐MCBS grading was used to assess the clinical significance of the RCTs with significantly positive findings [7]. In a cross‐sectional study involving Phase 3 RCTs related to NSCLC, breast cancer, colorectal cancer, and pancreatic cancer, del Paggio et al. [9] applied the ESMO‐MCBS framework to 49.8% of the trials, which was similar to the 50.0% we achieved. Among the positive RCTs, del Paggio et al. found that only 31.2% met the ESMO‐MCBS threshold for clinical benefit, which was similar to the proportion reported in the current study (27.6%) and in another study (36.4% of the RCTs related to gastric or gastroesophageal junction adenocarcinoma [47]). All these findings suggest that “statistical significance” is not equivalent to “clinical significance” [48]. The high expenses of novel anticancer drugs may present a great burden to the healthcare system, particularly for those that only provide limited benefits. In a recent study, Tibau et al. [49] applied ESMO‐MCBS grading to US Food and Drug Administration (FDA)‐approved molecular targeted drugs. Unexpectedly, of the 50 drugs covering 84 indications, only 24 (29%) met the threshold for clinical benefit. These results highlight the significance of identifying therapies with meaningful clinical benefits using the ESMO‐MCBS framework.

We found that more than half of the SCLC‐RCTs were designed to meet the ESMO‐MCBS benefit threshold. This “appropriate design” was more frequent in the superiority trials investigating first‐line treatment, indicating higher expectations of efficacy. Moreover, our results showed that the RCTs powered to identify an effect size that would meet the thresholds were more likely to demonstrate meaningful clinical benefit. This finding highlights the necessity of referring to the ESMO‐MCBS framework when planning power calculations for trials.

Generally, a strong endorsement of the experimental drug by the authors in RCTs was closely related to the statistical significance of the primary endpoint [50]. In the current study, RCTs satisfying the ESMO‐MCBS benefit threshold received stronger endorsement from the authors, which is in line with the study by del Paggio et al. [9]. However, only half of the strongly endorsed RCTs (8/16) demonstrated meaningful clinical benefits according to the ESMO‐MCBS grading. Trial investigators and journal editors should emphasize the importance of adequate and unbiased reporting of results.

Our results are affected by several limitations. First, some pivotal Phase 2 trials that provided key data for a new drug for regulatory approval may have been excluded from this study. Although these trials were more likely to present positive results, they typically represented only a small proportion [11]. Second, QoL was not evaluated in most studies, leading to a less accurate assessment of the magnitude of the clinical benefit. Third, the application of the ESMO‐MCBS framework to the trial design may be inaccurate owing to incomplete data, which requires further investigation.

SCLC‐RCTs significantly alter treatment approaches for the disease. Over the past 20 years, numerous innovative drug combinations have been investigated, among which platinum‐based chemotherapy combined with PD‐1/PD‐L1 inhibitors may be the most promising. However, the majority of the trials did not meet the ESMO‐MCBS benefit threshold, even for those “statistically significant” trials. Given the impact of statistical power on the true effect size, investigators and sponsors should refer to the ESMO‐MCBS framework when designing their statistical plans.

## Author Contributions

Yuejing Chen and Bo Cui conceived of the study and designed the study; Honghong Liu, Shaohua Bai, Xuejiao Han, and Fei Jin helped collect data; Yuejing Chen analyzed the data; Yuejing Chen wrote the manuscript; and Bo Cui helped revise the manuscript critically for important intellectual content. All authors read and approved the final manuscript.

## Ethics Statements

This was a retrospective review of publicly available data; the Ethics Committee of our center waived the requirement for ethics approval and informed consent.

## Conflicts of Interest

The authors declare no conflicts of interest.

## Supporting information


**Figure S1.** Forest plot showing the distribution of median overall survival of randomized controlled trials published during 2002–2023. # indicates trials that met the primary endpoint.
**Table S1.** ESMO‐Magnitude of Clinical Benefit Scale v1.1.
**Table S2.** Examples of scoring based on the ESMO‐MCBS framework.

## Data Availability

The datasets used and/or analyzed during the current study are available from the corresponding author on reasonable request.
