# Supplementary material for: Clinical Benefits of new Systemic Therapy for Small‐Cell Lung Cancer Over Two Decades: A Cross‐Sectional Study
Source: Clin Respir J. 2024 Oct 30;18(11):e70032. doi: 10.1111/crj.70032 (PMC11524636; doi:10.1111/crj.70032)
Supplement: Supplementary file 1 — Figure S1. Forest plot showing the distribution of median overall survival of randomized controlled trials published during 2002–2023. # indicates trials that met the primary endpoint. Table S1. ESMO‐Magnitude of Clinical Benefit Scale v1.1. Table S2. Examples of scoring based on the ESMO‐MCBS framework. [file CRJ-18-e70032-s001.docx]

**Figure S1.** Forest plot showing the distribution of median overall survival of randomized controlled trials published during 2002-2023. # indicates trials that met the primary endpoint.


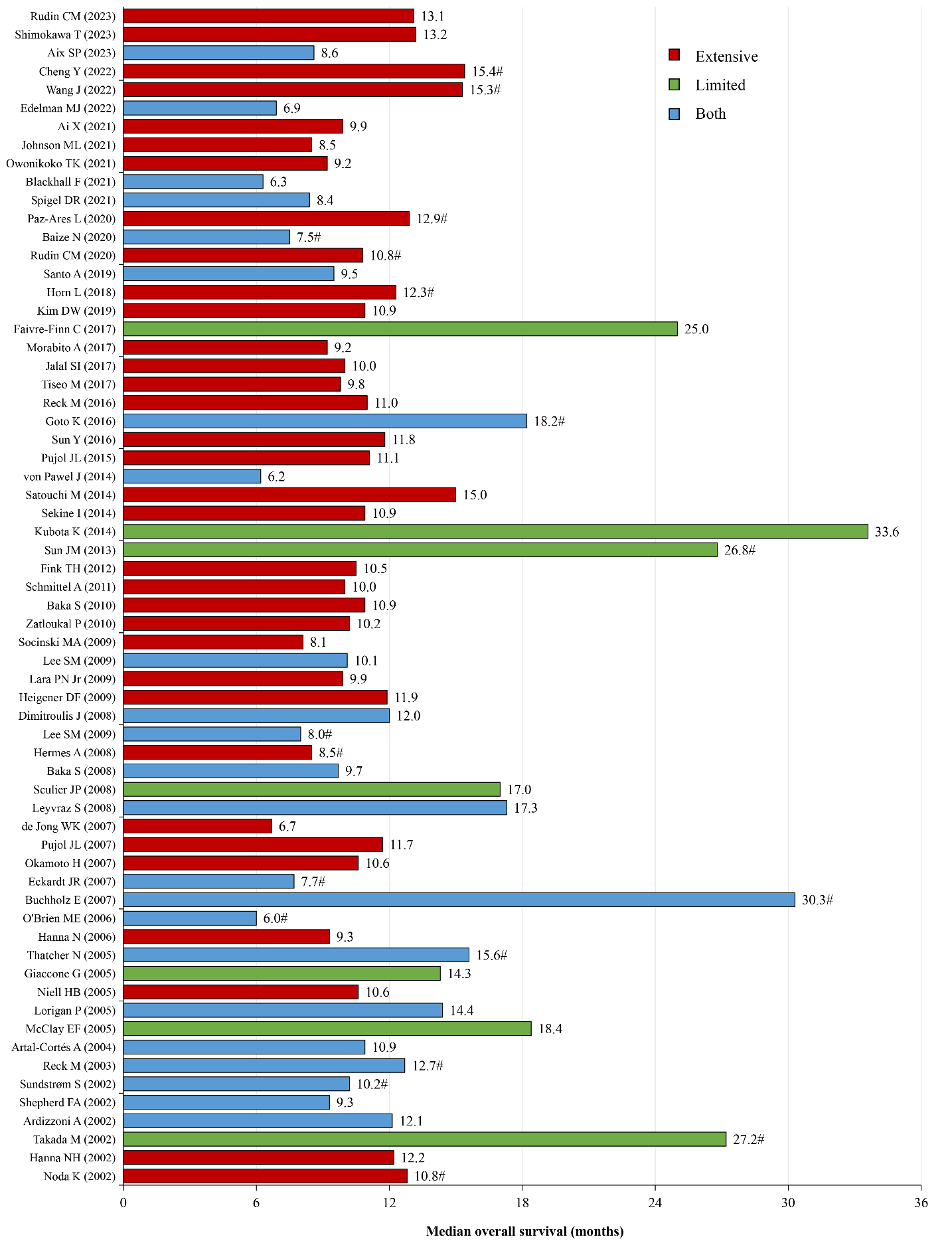


**Table S1.** ESMO-Magnitude of Clinical Benefit Scale v1.1.

| **Form 2a: for therapies that are not likely to be curative with primary endpoint of OS** | | | |
| --- | --- | --- | --- |
| **Grade** | **Median OS with the standard treatment** | | |
|  | ≤12 m | >12 m and ≤24 m | >24 m |
| 4 | HR ≤0.65 AND Gain ≥3 m  Increase in 2-y survival ≥10% | HR ≤0.70 AND Gain ≥5 m  Increase in 3-y survival ≥10% | HR ≤0.70 AND Gain ≥9 m  Increase in 5-y survival ≥10% |
| 3 | HR ≤0.65 AND Gain 2-3 m | HR ≤0.70 AND Gain ≥3, <5 m | HR ≤0.70 AND Gain ≥6, <9 m |
| 2 | HR ≤0.65 AND Gain 1.5-2 m  HR >0.65-0.70 AND Gain ≥1.5 m | HR ≤0.70 AND Gain 1.5-3 m  HR >0.70-0.75 AND Gain ≥1.5 m | HR ≤0.70 AND Gain 4-6 m  HR >0.70-0.75 AND Gain ≥4 m |
| 1 | HR >0.70 OR Gain <1.5 m | HR >0.75 OR Gain <1.5 m | HR >0.75 OR Gain <4 m |
| **Adjustments** | | | |
| A.Toxicity/QoL | Upgrade 1 level if improved QoL and/or less grade 3-4 toxicities impacting daily well-being. | | |
| B.Long-term Survival | If there is a long term plateau in the survival curve and OS advantage continues to be observe at 5 (Median OS with the standard treatment ≤12 m) or 7 years (Median OS with the standard treatment >12 m), also score according to form 1 (treatments with curative potential) and present boths cores i.e. A/4 | | |
| **Final adjusted magnitude of clinical benefit grade** 5 4 3 2 1 | | | |

| **Form 2b: for therapies that are not likely to be curative with primary endpoint of PFS** | | |
| --- | --- | --- |
| **Grade** | **Median PFS with the standard treatment** | |
|  | ≤6 m | >6 m |
| 3 | HR ≤0.65 AND Gain ≥1.5 m | HR ≤0.65 AND Gain ≥3 m |
| 2 | HR ≤0.65 AND Gain <1.5 m | HR ≤0.65 AND Gain <3 m |
| 1 | HR >0.65 | HR >0.65 |
| **Adjustments** | | |
| A.OS | When OS as secondary endpoint shows improvement, it will prevail and the new scoring will be done  according to form 2a | |
| B.Toxicity/QoL | Downgrade 1 level if there is one or more of the following incremental toxicities associated with the new  drug (toxic death >2%; cardiovascular ischemia >2%; hospitalization for toxicity >10%; excess rate of severe CHF >4%; grade 3 neurotoxicity >10%; severe other irreversible or long lasting toxicity >2%) | |
| C.OS | Downgrade 1 level if the drug only leads to improved PFS (mature data shows no OS advantage) and  QoL assessment does not demonstrate improved QoL | |
| D.Toxicity/QoL | Upgrade 1 level if improved QoL or if less grade 3-4 toxicities that bother patients are demonstrated | |
| E.Crossover | Upgrade 1 level if study had early crossover because of early stopping or crossover based on detection of survival advantage at interim analysis | |
| F. Long-term Survival | Upgrade 1 level if there is a long term plateau in the PFS curve, and there is >10% improvement in PFS at 1year | |
| **Final adjusted magnitude of clinical benefit grade** 4 3 2 1 | | |

| **Form 2c: for therapies that are not likely to be curative with primary endpoint other than OS or PFS or equivalence studies** | |
| --- | --- |
| **Grade** | **Primary outcome is Toxicity or QoL AND Non-inferiority Studies** |
| 4 | Reduced toxicity or improved QoL (using validated scale) with evidence for statistical non-inferiority or superiority in PFS/OS |
| 3 | Improvement in some symptoms (using a validated scale) BUT without evidence of improved overall QoL |
|  | **Primary outcome is Response Rate** |
| 2 | RR is increased >20% but no improvement in toxicity/QoL/PFS/OS |
| 1 | RR is increased <20% but no improvement in toxicity/QoL/PFS/OS |
| **Final magnitude of clinical benefit grade** 4 3 2 1 | |

**Table S2.** Examples of scoring based on the ESMO-MCBS framework.

| **Medication** | **Setting** | **Primary endpoint** | **PFS  control** | **PFS  gain** | **PFS  HR (95% CI)** | **OS  control** | **OS  gain** | **OS  HR (95% CI)** | **ORR  control** | **ORR gain** | **QoL/Toxicity** | **Adjustment** | **ESMO- MCBS** |
| --- | --- | --- | --- | --- | --- | --- | --- | --- | --- | --- | --- | --- | --- |
| **IMpower133**  Atezolizumab plus chemotherapy versus placebo plus chemotherapy | First-line | OS and PFS | 4.3 m | 0.9 m | 0.77 (0.63-0.95) | 10.3 m | 2.0 m | 0.76 (0.60-0.95) | 64.4% | -4.2% | Comparable toxicities and global QoL |  | 3  (form 2a) |
| **GFPC 01-2013** Carboplatin plus etoposide  versus topotecan | Second-line | PFS | 2.7 m | 2.0 m | 0.57 (0.41-0.73) | 7.4 m | 0.1 m | 1.03 (0.87-1.19) | 25% | 24% | Comparable toxicities, immature QoL | Fail to improve OS (down 1) | 2  (form 2b) |
| Topotecan plus cisplatin versus etoposide plus cisplatin | First-line | OS | NE | NE | NE | 40.9 w | 4.0 w | 0.93 (0.79-1.10) | 45.5% | 10.0% | Comparable toxicities |  | 1 (form 2c) |
| **Abbreviations:** PFS, progression-free survival; OS, overall survival; ORR, objective response rate; HR, hazard ratio; CI, confidence interval; QoL, quality of life; NE, not evaluable. | | | | | | | | | | | | | |
